# Long-term outcomes of adults with FSGS in the German Chronic Kidney Disease cohort

**DOI:** 10.1093/ckj/sfae131

**Published:** 2024-04-27

**Authors:** Eleni Stamellou, Jennifer Nadal, Bruce Hendry, Alex Mercer, Wibke Bechtel-Walz, Mario Schiffer, Kai-Uwe Eckardt, Rafael Kramann, Marcus J Moeller, Jürgen Floege, GCKD study investigators, Kai-Uwe Eckardt, Heike Meiselbach, Markus P Schneider, Mario Schiffer, Hans-Ulrich Prokosch, Barbara Bärthlein, Andreas Beck, André Reis, Arif B Ekici, Susanne Becker, Ulrike Alberth-Schmidt, Sabine Marschall, Anke Weigel, Gerd Walz, Anna Köttgen, Ulla T Schultheiß, Fruzsina Kotsis, Simone Meder, Erna Mitsch, Ursula Reinhard, Jürgen Floege, Turgay Saritas, Elke Schaeffner, Seema Baid-Agrawal, Kerstin Theisen, Kai Schmidt-Ott, Martin Zeier, Claudia Sommerer, Mehtap Aykac, Gunter Wolf, Martin Busch, Andi Steiner, Thomas Sitter, Christoph Wanner, Vera Krane, Britta Bauer, Florian Kronenberg, Julia Raschenberger, Barbara Kollerits, Lukas Forer, Sebastian Schönherr, Hansi Weissensteiner, Peter Oefner, Wolfram Gronwald, Matthias Schmid, Jennifer Nadal

**Affiliations:** Division of Nephrology and Clinical Immunology, RWTH Aachen University Hospital, Aachen, Germany; Department of Nephrology, School of Medicine, University of Ioannina, Ioannina, Greece; Department of Medical Biometry, Informatics and Epidemiology, Faculty of Medicine, University Hospital Bonn, Bonn, Germany; Travere Therapeutics , Inc., San Diego, CA, USA; JAMCO Pharma Consulting, Enskede, Sweden; Department of Medicine IV, University Medical Center, Faculty of Medicine, University of Freiburg, Freiburg im Breisgau, Germany; Berta‐Ottenstein Program, Faculty of Medicine, University of Freiburg, Freiburg im Breisgau, Germany; Department of Nephrology and Hypertension, Friedrich-Alexander Universität Erlangen-Nürnberg, Erlangen, Germany; Department of Nephrology and Hypertension, Friedrich-Alexander Universität Erlangen-Nürnberg, Erlangen, Germany; Department of Nephrology and Medical Intensive Care, Charité-Universitätsmedizin Berlin, Berlin, Germany; Division of Nephrology and Clinical Immunology, RWTH Aachen University Hospital, Aachen, Germany; Division of Nephrology and Clinical Immunology, RWTH Aachen University Hospital, Aachen, Germany; Division of Nephrology and Clinical Immunology, RWTH Aachen University Hospital, Aachen, Germany; Department of Cardiology, RWTH Aachen University Hospital, Aachen, Germany

**Keywords:** albuminuria, focal segmental glomerulosclerosis (FSGS), GCKD, kidney failure, outcomes

## Abstract

**Background:**

Focal segmental glomerulosclerosis (FSGS) can lead to kidney failure in adults. This study examines the progression of FSGS in the German Chronic Kidney Disease (GCKD) cohort.

**Methods:**

The GCKD study (*N* = 5217), a prospective cohort, included 159 patients with biopsy-confirmed FSGS recruited from 2010 to 2012. Baseline was defined as the first study visit. Adjudicated endpoints included a composite kidney endpoint (CKE), including an estimated glomerular filtration rate (eGFR) decrease >40%, eGFR <15 ml/min/1.73 m^2^ or initiation of kidney replacement therapy and combined major adverse cardiovascular events (MACE), including non-fatal myocardial infarction or stroke and all-cause mortality. Associations between baseline demographics, laboratory data, comorbidity and CKE and MACE were analysed using the Cox proportional hazards regression model.

**Results:**

The mean age at baseline was 52.1 ± 13.6 years, with a disease duration of 4.72 years (quartile 1: 1; quartile 3: 6) before joining the study. The median urinary albumin:creatinine ratio (UACR) at baseline was 0.7 g/g (IQR 0.1;1.8), while mean eGFR was 55.8 ± 23 ml/min/1.73 m^2^. Based on clinical and pathological features, 69 (43.4%) patients were categorized as primary FSGS, 55 (34.6%) as secondary FSGS and 35 (22%) as indeterminate. Over a follow-up of 6.5 years, 44 patients reached the composite kidney endpoint and 16 individuals had at least one MACE. UACR ≥0.7 g/g was strongly associated with both the composite kidney endpoint {hazard ratio [HR] 5.27 [95% confidence interval (CI) 2.4–11.5]} and MACE [HR 3.37 (95% CI 1.05–10.82)] compared with <0.7 g/g, whereas a higher eGFR at baseline (per 10 ml/min) was protective for both endpoints [HR 0.8 (95% CI 0.68–0.95) and HR 0.63 (95% CI 0.46–0.88), respectively]. Patients with secondary FSGS experienced a greater rate of eGFR decline than patients with primary FSGS.

**Conclusions:**

Lower eGFR and higher albuminuria are key risk factors for kidney disease progression and cardiovascular events in patients with FSGS.

KEY LEARNING POINTS **What was known:**

- Classification based on clinicopathological criteria is essential for determining focal segmental glomerulosclerosis (FSGS) aetiology and appropriate therapy.
- Studies show an increasing incidence of FSGS globally, but there is uncertainty about how much factors like proteinuria and body mass index affect long-term outcomes.

**This study adds:**

- This is the first study to examine the association of urinary albumin:creatinine ratio (UACR; as opposed to proteinuria) and kidney disease progression using adjudicated endpoints in FSGS patients. We found that higher levels of UACR were associated with an increased risk of both a composite kidney endpoint and a composite cardiovascular endpoint.
- We report a higher primary:secondary FSGS ratio than previously observed.
- We observed a more rapid estimated glomerular filtration rate (eGFR) decline in secondary FSGS compared with primary FSGS.

**Potential impact:**

- This study emphasizes the importance of UACR as a predictor of kidney disease progression and cardiovascular outcomes in FSGS patients.
- The observation of a faster eGFR decline in secondary FSGS could prompt a review of therapeutic approaches and more aggressive management strategies for secondary FSGS to slow disease progression.

## INTRODUCTION

Focal segmental glomerulosclerosis (FSGS) is not a specific disease entity, but rather a histological pattern of glomerular injury. The FSGS lesion has heterogeneous causes and can be subdivided into primary (‘idiopathic’) and secondary types. Secondary FSGS includes virus- and drug-associated FSGS, FSGS lesions superimposed on other glomerular diseases and maladaptive forms [1]. Maladaptive FSGS results from nephron loss or abnormal haemodynamic stress on a normal nephron population. Classification based on clinicopathological criteria is essential for determining appropriate therapy [2].

In several regions of the world, the incidence of FSGS has risen in recent decades [3]. Previous retrospective cohort studies often lacked clear definitions for different FSGS forms and yielded variable results for factors influencing long-term kidney outcomes. Notably, associations between long-term kidney outcomes and factors such as sex, baseline proteinuria, serum creatinine level and body mass index (BMI) at diagnosis remain inconclusive [4–9].

Observational studies and meta-analyses of randomized controlled trials (RCTs) have consistently established robust associations between albuminuria levels and the risk of kidney failure [10–13]. However, these studies encompass chronic kidney disease (CKD) in a broader context and are not specific for FSGS. Findings from FSGS cohorts suggest that proteinuria can be used as a surrogate for adverse outcome, as reductions in proteinuria are consistently associated with lower rates of kidney disease progression and failure [14–16]. However, there are no data regarding the use of albuminuria as a surrogate for kidney outcomes in FSGS.

The present study had two primary objectives. First, we aimed to investigate the association between baseline and time-dependent albuminuria and the risk of cardiovascular and kidney outcomes in a population with highly granular data and adjudicated endpoints by studying patients with an FSGS diagnosis enrolled in the German Chronic Kidney Disease (GCKD) cohort [17, 18]. Our second aim was to separately analyse the clinical characteristics and outcomes of primary and secondary FSGS by categorizing patients based on clinicopathological criteria.

## MATERIALS AND METHODS

### Study design and population

Between 2010 and 2012, the GCKD study enrolled 5217 participants of European ancestry 18–74 years of age with an eGFR of 30–60 ml/min/1.73 m^2^ or an eGFR ≥60 ml/min/1.73 m^2^ in the presence of increased albuminuria in a spot urine test [i.e. urine albumin:creatinine ratio (UACR) >300 mg/g creatinine]. The main exclusion criteria were non-European ancestry, active malignancy in the previous 2 years, previous transplantations or New York Heart Association class IV heart failure. A total of 159 participants with biopsy-proven FSGS were among study participants and included in the current analysis. The baseline date was defined as the first study visit and the follow-up time was 6.5 years.

Every participant in the study provided written informed consent. The ethics committees of all nine participating German institutions approved the study. The study was carried out in accordance with approved guidelines and the Declaration of Helsinki. The reporting guidelines of the Strengthening the Reporting of Observational Studies in Epidemiology were followed. The study was registered at the German Clinical Trials Register (www.drks.de; ID DRKS00003971).

### Outcomes

Adjudicated endpoints included a composite kidney endpoint (CKE) consisting of an eGFR decrease >40%, eGFR <15 ml/min/1.73 m^2^ or initiation of kidney replacement therapy (KRT), as well as the individual components of the kidney endpoint and combined major adverse cardiac events (MACE), including non-fatal myocardial infarction, stroke, cardiovascular death and all-cause mortality (death from a cardiovascular cause, cerebrovascular cause, peripheral vascular cause, infection, other cause, unknown cause or renal disease).

### FSGS aetiology

**Figure 1:**
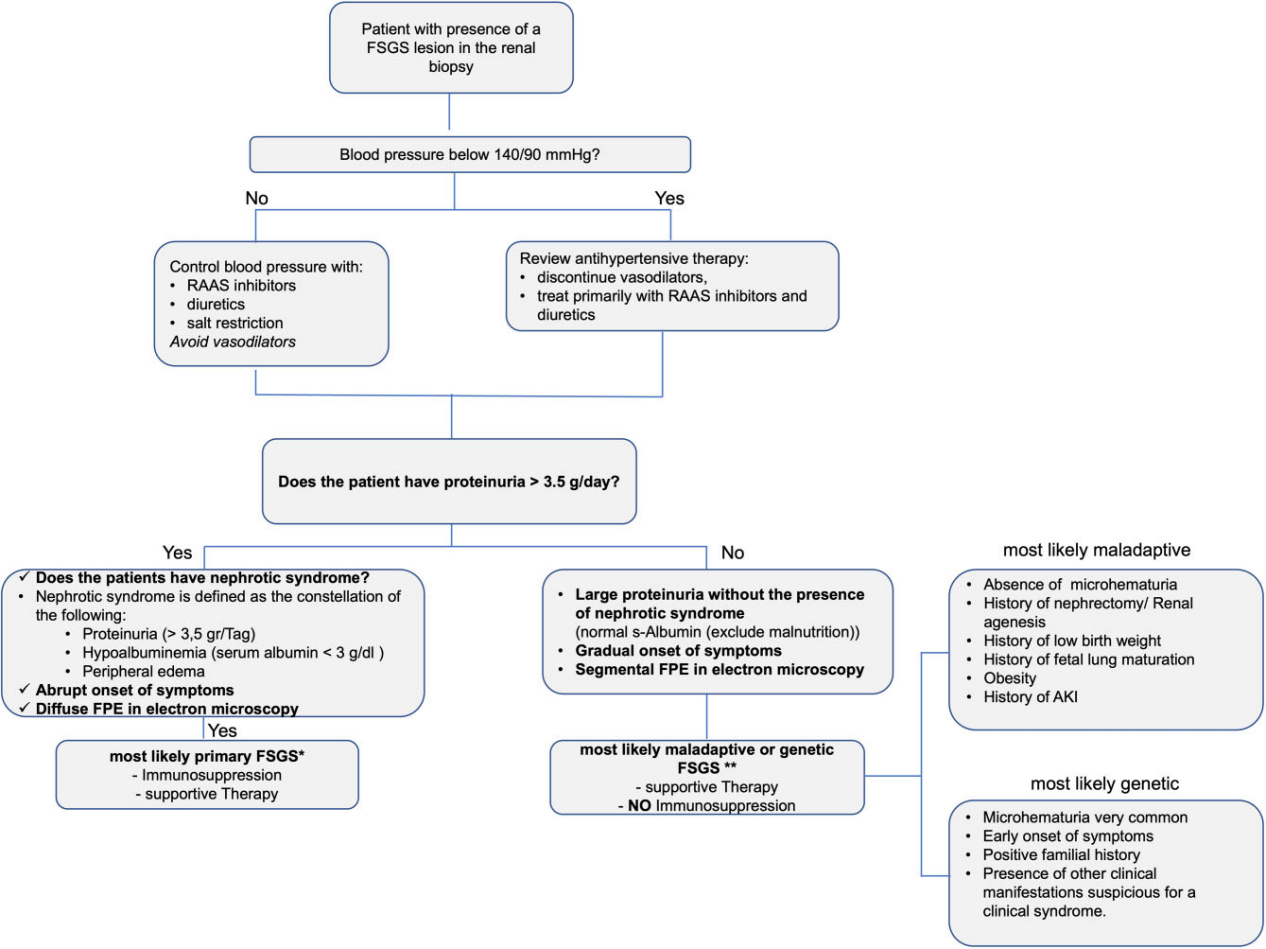
Proposed diagnostic algorithm for patients with FSGS based on De Vriese *et al.* [2]. RAAS: renin–angiotensin–aldosterone system; FPE: foot process effacement; AKI: acute kidney injury.

The categories of FSGS encompass four types: primary, secondary (including drug- or virus-induced and maladaptive), genetic and of undetermined cause. With the exception of primary FSGS, all others require supportive therapy rather than immunosuppression. This category encompasses patients with subnephrotic proteinuria and partial foot process effacement, and with no obvious FSGS cause. FSGS classification was based on clinical and laboratory information extracted from medical reports and kidney biopsy findings as well as a questionnaire (Supplementary Fig. 1) about the patient's medical history and disease course, employing proposed clinicopathological criteria [2]. According to these criteria, we developed a diagnostic algorithm to categorize patients (Fig. 1). Secondary forms included mainly those with maladaptive responses and one genetic case of FSGS requiring and treated with supportive therapy. The classification was primarily based on electron microscopy (EM) findings and further refined based on clinical presentation and laboratory data at baseline as well as response to immunosuppression. This approach aimed to ensure the most accurate categorization possible given the constraints of the data available in the GCKD cohort database. We assessed EM findings in our diagnostic process, with EM data being available and evaluated for 85 patients. Patients with insufficient clinical or biopsy data were categorized as indeterminate.

Complete renal biopsy and/or medical reports were available for review in 153 (96.8%) patients with FSGS. The classification was independently performed by two experienced nephrologists.

### Statistical analysis

Continuous variables were expressed as mean and standard deviation (SD) in the case of normally distributed variables or median and interquartile range (IQR) for non-normally distributed variables. Categorical variables were expressed as numbers with percentages. UACR was divided into two categories (<median and ≥median) based on the baseline measurement.

All data were collected and managed using Askimed as a cloud-based web platform (https://www.askimed.com). Data extraction from Askimed was performed in February 2021. The eGFR slopes were evaluated in linear mixed effects models. Repeated measurements of a participant were included by random intercept and random slope term (= random effects). Here, the random intercept allows a participant-specific eGFR at baseline and the random slope reflects a participant-specific change in eGFR over time.

Time-to-first event outcomes were defined as follows: If patients failed to complete the 6-year follow-up period, censoring was performed at the time of the last follow-up, e.g. when participants were lost to follow-up or refused to further participate in the study. The Cox regression analyses for the specific outcomes (composite kidney endpoint or MACE) were conducted with a competing risks approach using cause-specific Cox regression. Confounding risk factors at baseline included age, sex, BMI, FSGS aetiology, eGFR [Chronic Kidney Disease Epidemiology Collaboration (CKD-EPI) equation] and as time-dependent UACR categories. Estimates are presented as hazard ratios (HRs) with 95% confidence intervals (CIs). Additionally, we created a forest plot depicting the HR per each FSGS aetiology in each model.

To identify relevant variables of eGFR levels, we used linear regression modelling, including the following baseline covariates in the model: age, gender, BMI at baseline, FSGS aetiology and UACR as time dependent variables and presented the estimate (β) with 95% CIs.

Analyses were performed using SAS version 9.4 (SAS Institute, Cary, NC, USA).

## RESULTS

### Baseline demographic data

A total of 159 patients enrolled into the GCKD study had a diagnosis of FSGS, of which 69 (43.4%) were designated primary FSGS on clinicopathological criteria. The mean duration of the disease before enrolment was 2 years (quartile 1: 1; quartile 3: 6) (Supplementary Table 1). At baseline, the mean age was 52.1 ± 13.6 years, median albuminuria was 0.7 g/g (quartile 1: 0.1; quartile 3: 1.8) and the mean eGFR was 55.8 ± 23 ml/min/1.73 m^2^. Approximately 33% of the patients were in CKD stage G1 or G2 at baseline, about 50% in CKD stage G3 and 14% in CKD stage G4. The majority [101 (63.5%)] were male and nearly all of them had hypertension (99.4%). At baseline, 34% of the patients received treatment with corticosteroids while 23.3% were receiving immunosuppressive treatment other than steroids, with calcineurin inhibitors being the most common (Table 1).

**Table 1:** Demographics and clinical parameters at baseline stratified by FSGS aetiology.

Variable	Total (*N* = 159)	Primary [*n* = 69 (43.4%)]	Secondary [*n* = 55 (34.6%)]	Indeterminate [*n* = 35 (22%)]
Sex, *n* (%)				
Male	101 (63.5)	42 (60.9)	38 (69.1)	21 (60)
Female	58 (36.5)	27 (39.1)	17 (30.9)	14 (40)
Age (years), mean ± SD	52.1 ± 13.6	52.8 ± 13.3	51.2 ± 13.3	52.1 ± 15.1
BMI (kg/m^2^), mean ± SD	28.9 ± 5.6	29.2 ± 5.5	28.8 ± 5.4	28.4 ± 6.3
Systolic blood pressure (mmHg), mean ± SD	135.9 ± 19.4	132.3 ± 18.3	138.4 ± 21	139.5 ± 18
Diastolic blood pressure (mmHg), mean ± SD	80.4 ± 12.9	79.1 ± 11.3	81.6 ± 14.4	81.6 ± 13.6
Smoking, *n* (%)				
Non-smoker	67 (42.4)	31 (44.9)	23 (41.8)	13 (38.2)
Former smoker	54 (34.2)	27 (39.1)	16 (29.1)	11 (32.4)
Current smoker	37 (23.4)	11 (15.9)	16 (29.1)	10 (29.4)
UACR (g/g)	0.1;1.8	0.1;1.7	0.3;2.2	0.1;1.4
UACR categories, *n* (%)				
<0.7 g/g	83 (52.2)	37 (53.6)	25 (45.5)	21 (60)
≥0.7 g/g	76 (47.8)	32 (46.4)	30 (54.5)	14 (40)
eGFR (CKD-EPI) (ml/min/1.73 m^2^), mean ± SD	55.8 ± 23	60.4 ± 24.2	52.2 ± 21.9	52.2 ± 21.1
eGFR categories, *n* (%)				
G1: CKD-EPI ≥90	17 (10.8)	10 (14.7)	4 (7.3)	3 (8.8)
G2: CKD-EPI ≥60–<90	36 (22.9)	17 (25)	14 (25.5)	5 (14.7)
G3a: CKD-EPI ≥45–<60	50 (31.8)	23 (33.8)	14 (25.5)	13 (38.2)
G3b: CKD-EPI ≥30–<45	32 (20.4)	10 (14.7)	13 (23.6)	9 (26.5)
G4: CKD-EPI ≥15–<30	22 (14)	8 (11.8)	10 (18.2)	4 (11.8)
Diabetes, *n* (%)	36 (22.6)	22 (31.9)	7 (12.7)	7 (20)
Hypertension, *n* (%)	158 (99.4)	69 (100)	54 (98.2)	35 (100)
Coronary heart disease, *n* (%)	17 (10.7)	9 (13)	4 (7.3)	4 (11.4)
Antihypertensive medication, *n* (%)	156 (98.1)	68 (98.6)	53 (96.4)	35 (100)
ACEIs, *n* (%)	90 (56.6)	41 (59.4)	30 (54.5)	19 (54.3)
ARBs, *n* (%)	102 (64.2)	44 (63.8)	34 (61.8)	24 (68.6)
Diuretics, *n* (%)	98 (61.6)	46 (66.7)	31 (56.4)	21 (60)
Thiazides, *n* (%)	41 (25.8)	19 (27.5)	14 (25.5)	8 (22.9)
Loop diuretics, *n* (%)	66 (41.5)	36 (52.2)	18 (32.7)	12 (34.3)
Immunosuppressive treatment other than corticosteroids, *n* (%)	37 (23.3)	27 (39.1)	5 (9.1)	5 (14.3)
Systemic corticosteroids, *n* (%)	54 (34)	38 (55.1)	9 (16.4)	7 (20)
Dihydropyridine (nifedipine-type) , *n* (%)	57 (35.8)	20 (29)	23 (41.8)	14 (40)
Proton pump inhibitor, *n* (%)	53 (33.3)	28 (40.6)	14 (25.5)	11 (31.4)
Statin, *n* (%)	83 (52.2)	42 (60.9)	24 (43.6)	17 (48.6)
Acetylsalicylic acid, *n* (%)	36 (22.6)	16 (23.2)	12 (21.8)	8 (22.9)

### Kidney endpoints

**Figure 2:**
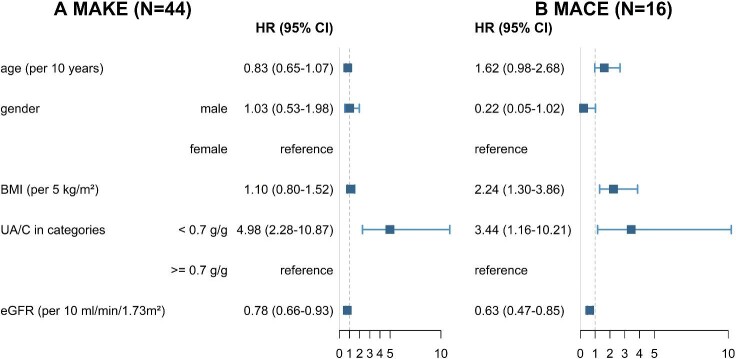
Forest plot of **(A)** CKE and **(B)** MACE. The forest plot shows HRs and 95% CIs of CKE and MACE.

**Figure 3:**
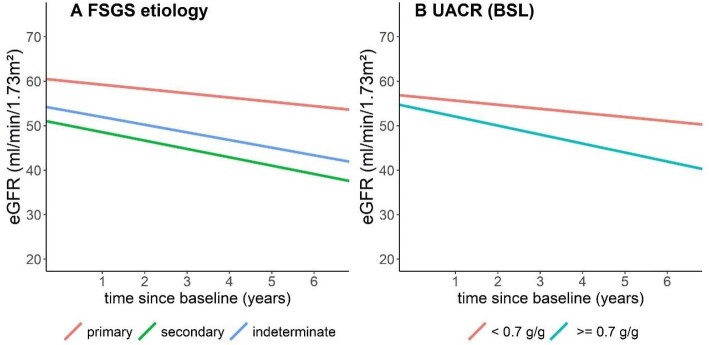
eGFR slopes according to **(A)** FSGS aetiology and **(B)** baseline UACR (g/g) over 6.5 years of follow-up.

During a follow-up of 6.5 years, 44 (27.7%) individuals reached the composite kidney endpoint, which included 19 (12%) who initiated KRT and an additional 25 (15.8%) who experienced a >40% eGFR decrease without initiating KRT. The univariate determinants of achieving the composite kidney endpoint were a change in UACR category, younger age, lower eGFR and prescription of a dihydropyridine calcium channel blocker (Supplementary Table 2). Participants with a UACR ≥0.7 g/g had a mean annual eGFR slope of −2.01, versus −0.92 for patients with a UACR <0.7 g/g (Fig. 3B, Supplementary Table 5). A multivariable Cox regression analysis revealed that a UACR ≥0.7 g/g compared with a lower UACR was associated with reaching the composite kidney endpoint [HR 5.27 (95% CI 2.4–11.5)]. In contrast, each 10 ml/min higher eGFR at baseline was found to be protective, with an HR of 0.80 (95% CI 0.68–0.95). Sex, age and BMI at baseline were not associated with the composite kidney endpoint in the multivariable analysis (Fig. 2A and Table 2).

**Table 2:** Multivariable Cox model for predictors of CKE and MACE.

Variables	CKE (*n* = 44/159), HR (95% CI)	MACE (*n* = 16/159), HR (95% CI)
Age per 10 years (BSL)	0.81 (0.63–1.04)	1.47 (0.84–2.58)
Sex		
Female	0.98 (0.5–1.91)	0.21 (0.04–1)
Male	Reference	Reference
BMI per 5 kg/m^2^ (BSL)	1.12 (0.81–1.55)	2.43 (1.34–4.39)
UACR categories		
≥0.7 g/g	5.27 (2.4–11.55)	3.37 (1.05–10.82)
<0.7 g/g	Reference	Reference
eGFR per 10 ml/min/1.73 m^2^ (BSL)	0.79 (0.66–0.93)	0.63 (0.46–0.88)
FSGS aetiology		
Indeterminate	1.44 (0.63–3.28)	
Secondary	0.74 (0.35–1.57)	0.64 (0.19–2.08)
Primary	Reference	Reference

BSL: baseline.

A linear mixed model for eGFR was used to assess the relationships with renal outcomes (Supplementary Table 3). The multivariable analysis revealed a negative association between age, with each 10-year increment linked to a more pronounced decline [estimate = −4.73 (95% CI −7.22 to −2.24)]. Moreover, FSGS aetiology demonstrated effects, with secondary FSGS associated with a more rapid decline in eGFR (Table 3).

**Table 3:** Multivariable linear mixed model analysis for baseline eGFR in a 6.5-year follow-up.

Effect	β	SE	95% CI
Age per 10 years (BSL)	−4.73	1.27	−7.22 to −2.24
Sex			
Female	−3.69	3.61	−10.77–3.39
Male	Reference		
BMI per 5 kg/m^2^ (BSL)	1.13	1.54	−1.89–4.15
UACR categories			
≥0.7 g/g	2.76	1.62	−0.41–5.94
≥0–<0.7 g/g	Reference		
FSGS aetiology			
Indeterminate	−7.88	4.53	−16.76–1.00
Secondary	−13.09	3.9	−20.73 to −5.45
Primary	Reference		−7.22 to −2.24

β: estimated coefficient; SE: standard error.

### Cardiovascular endpoints

During the follow-up period, 16 individuals experienced at least one MACE. A UACR ≥0.7 g/g was associated with experiencing a MACE (8 patients with non-fatal cardiovascular events, 8 patients with all-cause mortality), with an HR of 3.37 (95% CI 1.05–10.82) compared with <0.7 g/g. A higher eGFR at baseline (per 10 ml/min) was linked to a reduced risk of MACE, with an HR of 0.63 (95% CI 0.46–0.88). Additionally, a higher BMI at baseline (per 5 kg/m^2^) was also associated with an increased risk of MACE, with an HR of 2.43 (95% CI 1.34–4.39). Female sex provided a protective effect (Fig. 2B and Table 2).

### Outcomes per FSGS subtype

In our cohort, 69 (43.4%) patients were classified as having primary FSGS, 55 (34.6%) as having secondary FSGS and for 35 (22%) the aetiology was uncertain (Table 1). There were no differences in the baseline characteristics between the primary and secondary forms, except for immunosuppression, with nearly 55% of primary FSGS patients receiving corticosteroids and 40% receiving immunosuppression other than steroids at baseline, compared with 16.9% and 9%, respectively, for secondary FSGS (Table 1). Moreover, at baseline, diabetes was more common in patients with primary FSGS.

During a follow-up of 6.5 years, 15 (34.1%) individuals with primary, 17 (38.6%) with secondary and 12 (27.3%) with indeterminate FSGS reached the composite kidney endpoint (Supplementary Table 4). Similarly, no difference was found in the incidence of MACE among diagnostic groups (Supplementary Table 4).

The mean annual eGFR slope in primary FSGS was the smallest, at −0.96, followed by indeterminate at −1.73, and the secondary FSGS patients at −1.88 (Fig. 3A, Supplementary Table 5).

## DISCUSSION

The present study aimed to investigate the clinical characteristics and outcomes of patients diagnosed with FSGS who were enrolled in the GCKD cohort. A unique feature of our approach is that we attempted to classify patients into primary and secondary FSGS based on a clinicopathological approach and describe potential differences not only at baseline, but also during follow-up.

Traditionally, studies involving patients with FSGS have employed proteinuria, rather than albuminuria, as a surrogate for disease progression [6, 7, 9, 19]. To our knowledge, this study is the first to examine the association of UACR and kidney disease progression in FSGS patients. We found that higher levels of albuminuria were associated with an increased risk of both a composite kidney and a composite cardiovascular endpoint. It is important to note that in our cohort, baseline does not represent the time of diagnosis, but the first visit after enrolment in the GCKD study. The majority of our patients had been diagnosed several years earlier and had already received treatment. According to the recruitment strategy of the GCKD study, they were all under the care of a nephrologist. As all patients were on renin–angiotensin–aldosterone system inhibitor treatment and had reasonably well-controlled blood pressure, we can consider this FSGS subcohort as well-treated with respect to supportive measures. Consequently, our analysis demonstrates that the presence of a UACR >0.7 g/g is associated with an increased risk of kidney endpoints and MACE at any time during the course of the disease. Additionally, we confirm that a greater reduction in eGFR at baseline was associated with worse kidney survival rates [16, 20, 21].

Moreover, to the best of our knowledge, this is the first study directly comparing outcomes in patients with primary versus secondary FSGS. This categorization was performed retrospectively in a standardized way by two independent experienced nephrologists. Nevertheless, despite a rigorous definition of criteria for the categorization, this potentially led to misclassification. However, the higher frequency of immunosuppression at baseline in the primary FSGS group implies a largely correct classification. Moreover, survival bias should be considered, as it is possible that patients with more severe forms of primary FSGS were not included in the study. Such survival bias could also partially explain the slower rate of loss of eGFR in this cohort compared with studies where data from the diagnosis were analysed (e.g. the UK RaDaR FSGS cohort).

Furthermore, we report the relative disease frequencies of primary and secondary FSGS rather than reporting FSGS as a single disease entity. Within our cohort, we noted a substantial increase in the frequency of primary FSGS compared with previous findings [22]. However, it remains uncertain whether this represents a true increase in disease incidence, improved and earlier patient identification [23] or selection bias during recruitment into the GCKD cohort. Moreover, it can be argued that in a relatively high number of individuals, the form of FSGS could not be identified, potentially affecting the accuracy of reported incidence rates. Even if we assume that all these patients have a secondary form of FSGS, the incidence of primary FSGS (43%) is still high when compared with previous reports, in which primary FSGS accounted for roughly 25% of FSGS cases. Notably, in our analysis, patients with secondary FSGS did experience a more rapid decline in eGFR compared with those with primary FSGS. This could be attributed to the fact that we analysed the patients far later than disease onset and primary FSGS patients may already have received treatment. On the other hand, patients categorized with primary FSGS had lower levels of proteinuria and higher eGFRs, suggesting that they may be in remission.

Another interesting observation in our cohort is the higher frequency of diabetes in patients classified as having primary FSGS. This could be a side effect of long-term treatment protocols with high-dose steroids in these patients and warrants further investigation questioning the safety of the current treatment strategies.

Exposure to dihydropyridine calcium channel blockers was associated with worse renal survival in the univariate analysis, but these effects were not significant in a multivariable analysis. The lack of attenuation of CKD progression by dihydropyridine calcium channel blockers may be due to their association with increased albuminuria, even in patients under angiotensin-converting enzyme inhibitor (ACEI)/angiotensin II receptor blocker (ARB), possibly through dilation of the glomerular afferent arteriole [21]. Potential explanations for not confirming the effects of this medication in the multivariable model may be that our analyses do not account for the duration and intensity of therapy and the interaction with the severity of albuminuria. Another possible explanation could be that patients treated with dihydropyridine calcium channel blockers in the context of more severe or poorly controlled hypertension were sicker patients who required additional antihypertensive therapy. This could explain why the significance of the association is lost in the multivariable analysis when adding cofounding variables such as albuminuria, BMI and eGFR.

A major limitation of our study is that follow-up data may be incomplete from disease onset or diagnosis. Second, despite the large size of the GCKD cohort, our sample size was relatively small and limited to people of European ancestry, which may influence the statistical power and generalizability of our results. The homogeneous ethnic background of our study population diminishes the influence of genetic factors. The participants were enrolled in Germany on the basis of prevalent non-dialysis-dependent CKD, thus there is a selection bias of participants owing to this defined study cohort. Further larger-scale studies with longer follow-up periods are warranted to validate our findings and further elucidate the factors influencing the prognosis of FSGS patients. In our study, serum albumin levels at the time of disease diagnosis were not uniformly available for all patients. Where these data were accessible, they were incorporated into our analysis and contributed to the classification of FSGS cases. It is important to acknowledge that the absence of consistent baseline serum albumin levels across the patient cohort represents a limitation of our study. Despite this, the available albumin data provided valuable insights into the clinical status of the patients at the time of diagnosis. Further limitations include the lack of comprehensive genetic testing data, which could lead to misclassification within the secondary and unknown categories of FSGS. The heterogeneity of these groups and the variable clinical presentations of genetic FSGS underscore the need for cautious interpretation of our findings. In analysing the outcomes of this cohort, we acknowledge that differences in treatment may confound the interpretations. The range of interventions, from immunosuppressive regimens to supportive care, reflects the real-world complexity of managing FSGS but also poses a challenge in attributing outcomes to specific aetiologies or disease stages. Finally, we acknowledge that pooling together cases of complete remission, partial remission, steroid-resistant and steroid-dependent FSGS complicates interpretation of our data.

Strengths of our analysis include that the cohort exclusively consists of patients treated by nephrologists, ensuring relatively standardized care. This not only minimizes potential cofounding variables arising from variations in medical management, but also highlights the clinical relevance of our results. Second, the GCKD is a cohort with uniquely granular data related to patient demographics, medical history, treatment regimens and outcomes. Systematic endpoint adjudication further enhanced outcome accuracy and reduced bias. Moreover, we used standardized questionnaires to evaluate participants’ characteristics and in-person study visits conducted by trained study nurses. Outcomes were continuously evaluated by experienced physicians according to predefined criteria in a standardized fashion based on hospital discharge letters and death certificates.

In summary, our study identifies low eGFR and, in particular, a higher UACR as potent predictors of adverse kidney and cardiovascular outcomes. Patients with secondary FSGS experienced a faster eGFR decline compared with primary FSGS, emphasizing the impact of secondary FSGS on kidney outcomes and the need for intensified supportive therapy in this subgroup.

## Supplementary Material

sfae131_Supplemental_File

## Data Availability

The data underlying this article are available in the article and in its online supplementary material.

## APPENDIX 1

Current GCKD investigators and collaborators with the GCKD study: University of Erlangen-Nürnberg: Kai-Uwe Eckardt, Heike Meiselbach, Markus P. Schneider, Mario Schiffer, Hans-Ulrich Prokosch, Barbara Bärthlein, Andreas Beck, André Reis, Arif B. Ekici, Susanne Becker, Ulrike Alberth-Schmidt, Sabine Marschall, Anke Weigel; University of Freiburg: Gerd Walz, Anna Köttgen, Ulla T. Schultheiß, Fruzsina Kotsis, Simone Meder, Erna Mitsch, Ursula Reinhard; RWTH Aachen University: Jürgen Floege, Turgay Saritas; Charité, University Medicine Berlin: Elke Schaeffner, Seema Baid-Agrawal, Kerstin Theisen; Hannover Medical School: Kai Schmidt-Ott; University Hospital, Renal Center, Heidelberg: Martin Zeier, Claudia Sommerer, Mehtap Aykac; University Hospital Jena: Gunter Wolf, Martin Busch, Andi Steiner; Ludwig-Maximilians University of München: Thomas Sitter; University of Würzburg: Christoph Wanner, Vera Krane, Britta Bauer; Medical University of Innsbruck, Division of Genetic Epidemiology: Florian Kronenberg, Julia Raschenberger, Barbara Kollerits, Lukas Forer, Sebastian Schönherr, Hansi Weissensteiner; University of Regensburg, Institute of Functional Genomics: Peter Oefner, Wolfram Gronwald; Department of Medical Biometry, Informatics and Epidemiology, University Hospital of Bonn: Matthias Schmid, Jennifer Nadal.
