# Supplementary material for: Long-term outcomes of adults with FSGS in the German Chronic Kidney Disease cohort
Source: Clin Kidney J. 2024 Apr 27;17(7):sfae131. doi: 10.1093/ckj/sfae131 (PMC11234294; doi:10.1093/ckj/sfae131)

**Supplemental Material**

| **Suppl. Table 1: Disease duration at baseline** | | | | |
| --- | --- | --- | --- | --- |
| **Duration** | **N** | **Median** | **Q1** | **Q3** |
| TOTAL | 151 | 2 | 1 | 6 |
|  |  |  |  |  |
| primary | 69 | 2 | 1 | 6 |
| secondary | 53 | 1 | 1 | 5 |
| indeterminate | 29 | 3 | 2 | 9 |

| Suppl. Table 2: univariate cox model for predictors of CKE and MACE  (adjusted for UACR^TD^ in categories Ref: <0.7 g/g) | | | |
| --- | --- | --- | --- |
|  |  | CKE  44/159 | MACE  16/159 |
|  |  | HR [95% CI] | HR [95% CI] |
| Sex^BSL^ | female (Ref: male) | 1.43 [0.78; 2.61] | 0.5 [0.14; 1.77] |
| age (per 10 years)^BSL^ |  | 1 [0.8; 1.25] | 1.6 [1.03; 2.48] |
| BMI (per 5 kg/m²)^BSL^ |  | 1.04 [0.76; 1.41] | 1.42 [0.95; 2.12] |
| Systolic blood pressure (mmHg)^BSL^ |  | 1.02 [1; 1.03] | 1.02 [1; 1.05] |
| Diastolic blood pressure (mmHg)^BSL^ |  | 1.03 [1; 1.06] | 1 [0.96; 1.04] |
| eGFR (CKD-EPI) ml/min/1.73m^2^ |  | 0.85 [0.74; 0.98] | 0.62 [0.45; 0.84] |
| Diabetes^BSL^ | yes (Ref: no) | 0.86 [0.4; 1.86] | 1.72 [0.59; 4.97] |
| Coronary heart disease^BSL^ | yes (Ref: no) | 1.38 [0.58; 3.28] | 2.88 [0.81; 10.23] |
| Antihypertensive medication^BSL^ | yes (Ref: no) | 0.62 [0.08; 4.62] | 0.55 [0.07; 4.22] |
| ACE-Inhibitors^BSL^ | yes (Ref: no) | 1.3 [0.66; 2.55] | 1 [0.37; 2.7] |
| ARBs^BSL^ | yes (Ref: no) | 1.66 [0.88; 3.15] | 0.43 [0.15; 1.26] |
| Diuretics^BSL^ | yes (Ref: no) | 1.03 [0.55; 1.91] | 1.98 [0.63; 6.19] |
| Thiazides^BSL^ | yes (Ref: no) | 1.4 [0.7; 2.77] | 1.61 [0.56; 4.68] |
| Loop diuretics^BSL^ | yes (Ref: no) | 1.09 [0.6; 2] | 3.47 [1.19; 10.09] |
| Immunosuppressive Treatment other than corticosteroids^BSL^ | yes (Ref: no) | 0.99 [0.49; 2.03] | 0.91 [0.26; 3.26] |
| Systemic corticosteroids^BSL^ Glucocorticoide | yes (Ref: no) | 1.34 [0.53; 3.43] | 1.23 [0.41; 3.65] |
| Dihydropyridine (Nifedipin-Type)^BSL^ | yes (Ref: no) | 2.23 [1.21; 4.1] | 0.67 [0.21; 2.08] |
| Proton Pump Inhibitors^BSL^ | yes (Ref: no) | 1.52 [0.81; 2.82] | 1.33 [0.48; 3.67] |
| Statine^BSL^ | yes (Ref: no) | 1.49 [0.79; 2.79] | 0.94 [0.35; 2.55] |
| Acetylsalicylsäure^BSL^ | yes (Ref: no) | 1.17 [0.58; 2.38] | 0.79 [0.22; 2.78] |

| Suppl. Table 3: Univariate linear Mixed Model for baseline eGFR in a 6.5-Year Follow-Up (adjusted for UACR^TD^ in categories Ref: <0.7 g/g) | | |
| --- | --- | --- |
| Variable | | β [95% CI] |
| Sex^BSL^ | female (Ref: male) | -4.36 [-11.79; 11.22] |
| age (per 10 years)^BSL^ |  | -4.44 [-6.97; 3.82] |
| BMI (per 5 kg/m²)^BSL^ |  | 1.37 [-1.82; 4.82] |
| Systolic blood pressure (mmHg)^BSL^ |  | -0.21 [-0.39; 0.27] |
| Diastolic blood pressure (mmHg)^BSL^ |  | -0.18 [-0.45; 0.41] |
| Diabetes^BSL^ | yes (Ref: no) | 1.17 [-7.51; 13.11] |
| Hypertension^BSL^ | yes (Ref: no) | 7.51 [-36.84; 66.98] |
| Coronary heart disease^BSL^ | yes (Ref: no) | -12.81 [-24.10; 17.05] |
| Antihypertensive medication^BSL^ | yes (Ref: no) | -9.17 [-17.79; 13.02] |
| ACE-Inhibitors^BSL^ | yes (Ref: no) | 0.37 [-3.94; 6.51] |
| ARBs^BSL^ | yes (Ref: no) | -4.52 [-8.15; 5.48] |
| Diuretics^BSL^ | yes (Ref: no) | -6.51 [-10.08; 5.39] |
| Thiazides^BSL^ | yes (Ref: no) | -3.13 [-7.29; 6.28] |
| Loop diuretics^BSL^ | yes (Ref: no) | -8.83 [-12.75; 5.92] |
| Immunosuppressive Treatment other than corticosteroids^BSL^ | yes (Ref: no) | 0.97 [-4.03; 7.55] |
| Systemic corticosteroids^BSL^ Glucocorticoide | yes (Ref: no) | -3.7 [-7.64; 5.95] |
| Dihydropyridine (Nifedipin-Type)^BSL^ | yes (Ref: no) | -5.11 [-9.11; 6.04] |
| Proton Pump Inhibitors^BSL^ | yes (Ref: no) | -0.04 [-3.84; 5.74] |
| Statine^BSL^ | yes (Ref: no) | -3.46 [-7.79; 6.54] |
| Acetylsalicylsäure^BSL^ | yes (Ref: no) | -8.26 [-13.83; 8.41] |

BSL: baseline

| Suppl. Table 4: Incidence of MCE and MACE by FSGS subgroup | | |
| --- | --- | --- |
|  | MCE | MACE |
| Primary | 15 (34.1%) | 8 (50%) |
| Secondary | 17 (38.6%) | 8 (50%) |
| Indeterminate | 12 (27.3%) | 0 |

| Suppl. Table 5:eGCR slope | | | |
| --- | --- | --- | --- |
|  |  | Intercept | Slope |
| UACR categories | < 0.7 g/g | 56.56 | -0.92 |
|  | ≥ 0.7 g/g | 54.07 | -2.02 |
| FSGS etiology | Indeterminate | 53.67 | -1.73 |
|  | secondary | 50.42 | -1.88 |
|  | Primary | 60.18 | -0.96 |

**Suppl. Figure 1**


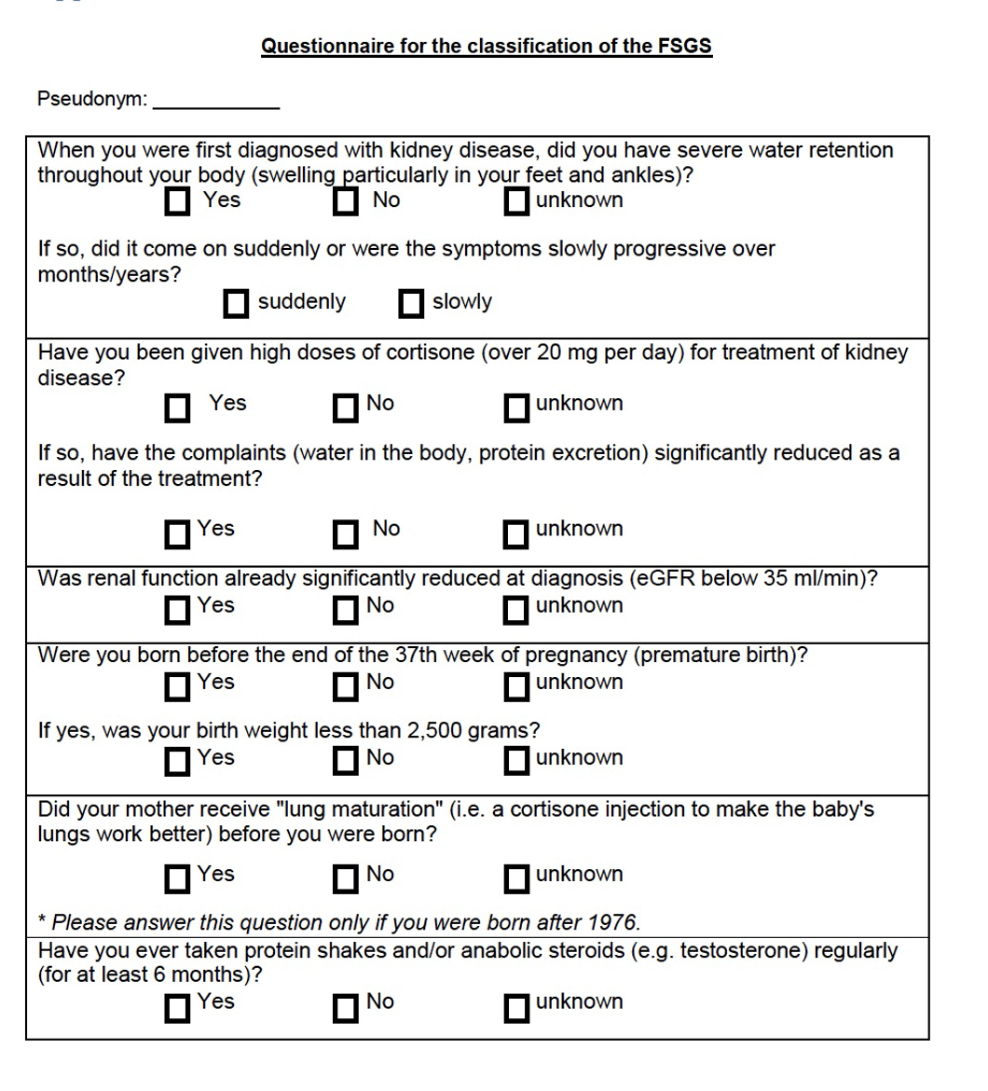

Supplement: sfae131_Supplemental_File [file sfae131_supplemental_file.docx]
